# A phase I study of lenalidomide plus chemotherapy with idarubicin and cytarabine in patients with relapsed or refractory acute myeloid leukemia and high‐risk myelodysplastic syndrome

**DOI:** 10.1002/ajh.25958

**Published:** 2020-09-19

**Authors:** Caner Saygin, Karilyn Larkin, James S. Blachly, Shelley Orwick, Apollinaire Ngankeu, Charles T. Gregory, Mitch A. Phelps, Shylaja Mani, Alison Walker, Ramiro Garzon, Sumithira Vasu, Katherine J. Walsh, Bhavana Bhatnagar, Rebecca B. Klisovic, Michael R. Grever, Guido Marcucci, John C. Byrd, William Blum, Alice S. Mims

**Affiliations:** ^1^ Department of Internal Medicine The Ohio State University Columbus Ohio; ^2^ Division of Hematology The Ohio State University Comprehensive Cancer Center Columbus Ohio; ^3^ Division of Pharmaceutics College of Pharmacy, The Ohio State University Columbus Ohio; ^4^ Department of Hematology and Medical Oncology Emory University School of Medicine, Winship Cancer Institute Atlanta Georgia; ^5^ Department of Hematology and Hematopoietic Cell Transplantation City of Hope Medical Center Duarte California

## Abstract

Patients with relapsed/refractory (R/R) acute myeloid leukemia (AML) have poor outcomes and hematopoietic cell transplantation (HCT) is the only curative treatment. New targeted therapies improved survival in select patients with specific mutations, however management of patients without these molecular alterations is an unmet need. We conducted a phase one study of lenalidomide in combination with cytarabine/idarubicin salvage chemotherapy in patients with R/R AML and high‐risk myelodysplastic syndromes. A total of 33 patients were enrolled in the study (30 AML, 3 MDS), and treated at three dose levels with 3 + 3 design. Dose‐limiting toxicity (DLT) was seen in eight patients, including four hematologic DLTs. The most commonly observed non‐hematologic serious adverse events were febrile neutropenia, rash, sepsis and renal injury. Dose level −1, consisting of 25 mg/d lenalidomide D1‐21, 1 g/m^2^ cytarabine D5‐8, and 8 mg/m^2^ idarubicin D5‐7 was determined to be the maximum tolerated dose. Note, 15/33 (45%) of patients were able to receive pre‐planned 21 days of lenalidomide. Overall, 18 patients achieved complete remission (CR) (n = 14) or CR with incomplete count recovery (CRi) (n = 4) with total CR/CRi rate of 56%. The 1‐year and 2‐year overall survival (OS) were 24% and 10%, respectively. Among responders, 10/18 underwent allogeneic HCT and had a 1‐year OS of 40%. There was no molecular pattern associated with response. These data demonstrate that the combination had clinical activity in R/R AML. This regimen should be further investigated for patients who relapsed after HCT, and as a bridge therapy to HCT. (ClinicalTrials.gov identifier: NCT01132586).

## 1 INTRODUCTION

The initial treatment for most fit patients with newly diagnosed acute myeloid leukemia (AML) consists of the combination of an anthracycline (daunorubicin or idarubicin) and cytarabine, which has led to remission rates approaching 60%‐80%.^1^ However, patients who do not respond to initial chemotherapy or experience relapse after initial complete remission (CR) have dismal outcomes.^2^ Hematopoietic cell transplantation (HCT) has curative potential in a minority of fit relapsed/refractory (R/R) AML patients who can achieve CR after salvage therapy or those with a low Duval score.^3^ Response rates with commonly used cytarabine‐based reinduction regimens (eg, mitoxantrone, etoposide, cytarabine [MEC] or fludarabine, cytarabine, filgrastim [FLAG]) can reach 40%‐60% if CR duration was 1 year or longer. But, this rate drops to 10%‐15% in cases with shorter CR duration and multiple lines of prior therapy.^4^ Therefore, new therapeutic strategies that are tolerable and more efficacious are needed to improve outcomes in R/R AML patients.

Lenalidomide is an immunomodulatory agent, approved by the US Food and Drug Administration for the management of multiple myeloma, myelodysplastic syndrome (MDS) with del(5q) and certain subtypes of lymphoma.^5^ The mechanism of action of lenalidomide is incompletely understood, but previous investigations proposed activation of innate and adaptive immune responses, altered cell cycling, disruption of marrow microenvironment, ubiquitination of critical targets and induction of expression of the tumor suppressor genes.^6^ Its unique clinical activity in MDS with del(5q) was shown to be associated with the degradation of casein kinase 1A1, resulting in death of malignant progenitor cells.^7^ In AML mouse models, lenalidomide treatment was shown to exhibit antitumorigenic activity through increased expression of *mirR‐181a*, which resulted in increased sensitivity to cytotoxic chemotherapy.^8^

We have previously demonstrated single agent activity of lenalidomide in patients with R/R AML.^9^ The dose was safely escalated to 50 mg daily for 21 days of a 28‐day cycle with a CR rate of 16% (5/31). Fatigue was the dose limiting toxicity (DLT) but remissions were durable, lasting 5 to 14 months. Remissions with full donor chimerism were also observed in post‐transplant patients, suggesting a possible immunomodulatory effect. Similarly, a recent study investigating the combination of lenalidomide with azacitidine in AML patients who relapsed after allogeneic HCT, reported a 47% (7/15) response rate after three cycles of treatment.^10^ The maximum tolerated dose (MTD) was determined as 25 mg daily, which did not reverse biologic features of Tcell exhaustion. When lenalidomide was combined with intermediate‐dose cytarabine in a phase one study of R/R AML patients, MTD for lenalidomide was 10 mg daily, overall response rate was 41% (13/32) with 16% (5/32) CR and 16% (5/32) CR with incomplete count recovery (CRi).^11^ Finally, lenalidomide in combination with MEC re‐induction therapy in R/R AML resulted in 34% (12/35) CR rate with MTD of 50 mg daily.^12^ The drug was also tested in the first‐line setting as a single agent or in combination with other chemotherapy regimens for previously untreated AML.^13^, ^14^, ^15^, ^16^, ^17^, ^18^, ^19^, ^20^, ^21^

Given the clinical activity of single agent lenalidomide in AML and preclinical studies suggestive of synergy with chemotherapy, we conducted a phase one study of lenalidomide in combination with cytarabine and idarubicin re‐induction chemotherapy, to evaluate the safety and tolerability of this combination in patients with R/R AML and high‐risk MDS.

## 2 METHODS

### 2.1 Eligibility criteria and study design

The study (NCT01132586) was approved by the Institutional Review Board of The Ohio State University, conducted in accordance with the Declaration of Helsinki, and all patients provided written informed consent. Eligible patients were adults aged 18 to 65 with a confirmed pathologic diagnosis of AML or high‐risk MDS which had recurred or was refractory to at least one prior line of therapy. Patients who could not attain CR after one round of induction therapy containing anthracycline plus cytarabine (100‐200 mg/m^2^ every 24 hours for 7 days) were considered to have refractory disease and were eligible for this study. High‐risk MDS was defined as International Prognostic Scoring System (IPSS) score of 1.5 or higher.^22^ Cytogenetic risk for AML patients was defined per European LeukemiaNet 2017 criteria.^23^ Patients with secondary AML and therapy‐related myeloid neoplasms were eligible. Patients were required to have an Eastern Cooperative Oncology Group (ECOG) performance status of zero to two, and adequate organ function including a total bilirubin <2 mg/dL, ALT and AST <2.5× upper limit of normal, left ventricular ejection fraction ≥40%, creatinine <2 mg/dL and calculated creatinine clearance ≥50 mL/min. Patients who had previously received lenalidomide, cytarabine and/or idarubicin were eligible provided that the combination of the three agents had never been administered, and that no lenalidomide had been administered for at least 6 months. Patients were excluded if they had active central nervous system disease, uncontrolled systemic infections, and if any chemotherapy or radiotherapy within 2 weeks prior to enrollment. All participants had to agree to practice appropriate contraception. Hydroxyurea was permitted until day 5 if necessary to maintain white blood cell count (WBC) <40 000/μL.

**FIGURE 1:**
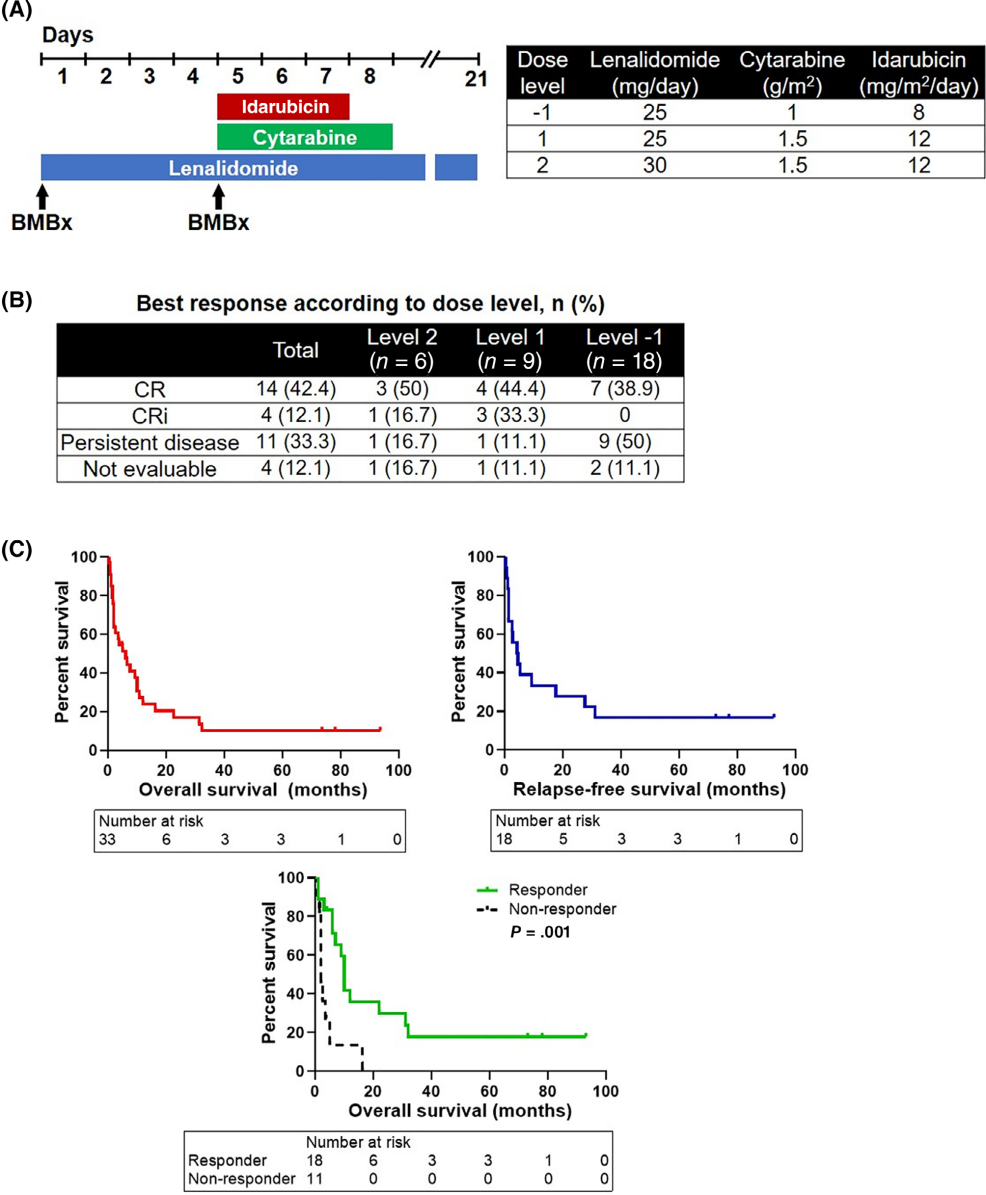
A, Study schema for 21‐day re‐induction cycle in relapsed/refractory acute myeloid leukemia or high‐risk myelodysplastic syndrome cohort. Patients underwent bone marrow (BM) biopsy prior to study entry and on day 5 after single‐agent lenalidomide therapy. Cytarabine was started on day 5 and administered as 96‐hour continuous infusion. Idarubicin (Ida) was given intravenously once a day on days 5, 6, 7 and each day the infusion lasts 1 hour. The table shows dosing schema for the study drugs at dose levels −1, 1 and 2. B, Best response to trial therapy according to dose level. CR, complete response; CRi, CR with incomplete count recovery. C, Kaplan–Meier overall survival and relapse‐free survival curves of all patients treated on this study, as well as comparison between patients who achieved remission on this study (responders) and patients who were refractory to study regimen (non‐responders) [Color figure can be viewed at wileyonlinelibrary.com]

Patients were treated with 21‐day course of oral lenalidomide in addition to 96‐hour continuous intravenous (IV) cytarabine infusion administered on days 5‐8 and idarubicin administered IV as 1‐hour infusion on days 5, 6 and 7 (Figure 1A). Lenalidomide dose was escalated in separate cohorts using a classic 3 + 3 phase one design to determine the MTD with this combination. No intrapatient dose escalation was allowed. After establishment of the MTD, a planned 12‐patient expansion group was included to better estimate the tolerability of this dose and to provide a preliminary efficacy assessment. All patients underwent bone marrow biopsy at baseline, on day 5 after single‐agent lenalidomide therapy, and on day 28 after the completion of induction cycle. Response was assessed according to the International Working Group criteria for AML and MDS.^24^, ^25^ Patients who achieved CR were evaluated for allogeneic HCT or a single cycle of consolidation therapy on this trial, which consisted of oral lenalidomide dosed as during the induction, cytarabine on days 5‐7, and idarubicin on days 5 and 6.

### 2.2 Safety assessment

Adverse events were graded per the Common Terminology Criteria for Adverse Events (version 4.0). The DLT was defined during cycle one of therapy. It is recognized that drug‐related toxicity in this population may be difficult to ascertain given the aggressive nature of the disease. Nonhematologic grade four toxicities were defined as DLTs if they were drug‐related. Toxicity attributed to any of the three agents was considered dose‐limiting. Hematologic DLT was defined as failure to recover absolute neutrophil count (ANC <500/μL) or platelet count (<25 000/μL) by day 42 in the absence of persistent disease. If the DLT occurred in two or more patients at a single dose level, then that dose was deemed intolerable and the next lower dose level was expanded. Given the high prevalence of infectious complications associated with cytopenias, infection‐related complications were not considered DLT unless severity or duration was longer than expected with conventional treatment. Alopecia, nausea and vomiting controllable with antiemetic therapy, line‐associated venous thrombosis, transient and correctable electrolyte abnormalities were also not considered DLT.

### 2.3 Mutational analysis

Viably preserved patient bone marrow and blood samples were sequenced using targeted amplicon sequencing on the HiSeq (Illumina) platforms to detect common myeloid mutations as described before.^26^ Genomic DNA was isolated from viably preserved patient bone marrow and blood samples using the QIAmp DNA Mini Kit (QIAGEN, Hilden, Germany). Genomic DNA fragmentation was done via focused ultrasonication (Covaris, Woburn, Massachusetts). The NGS libraries were prepared using a KAPA HyperPlus Kit (Roche, Pleasanton, CA). Target Enrichment was performed with xGen Lockdown Probes (IDT, Coralville, IA). Libraries were sequenced using the Illumina HiSeq 4000 (Illumina, San Diego, CA). Sequenced reads were aligned to the hg19 genome build using the Burrows‐Wheeler Aligner (BWA). Picard Tools was used to perform UMI‐consensus calling on the aligned reads. The Genome Analysis Toolkit (GATK) was used to realign insertions and deletions in the aligned reads and to perform base quality score recalibration for those realigned regions. Also GATKʼs MuTect2 was used to perform variant calling. After variant calling, variants were annotated using SnpEff and vcfanno along with the dbsnp, COSMIC, 1000 genomes, and 6500 exomes variant databases. The Mucor3 algorithm was used as the baseline for integrative mutation assessment. All called variants underwent visual inspection of the aligned reads using the Integrative Genomics Viewer. Variants in regions of high discrepancy, low quality, tandem repeats, or mononucleotide runs were excluded. Variants that were annotated as a likely germline variant by 1000 genomes or 6500 exomes and visually suspected of being germline were also removed. We used variant allelic frequency (VAF) ≥5% as cutoff to call a mutation. Both *NPM1* and *CEBPA* mutation results were confirmed in a clinical PCR/Sanger assay. The *FLT3*‐internal tandem duplications (*FLT3*‐ITD) were evaluated using capillary electrophoresis fragment analysis. Pretreatment and posttreatment samples were analyzed similarly and monitored for changes in VAF.

### 2.4 Outcomes and statistical analysis

The primary objective was to determine the MTD of lenalidomide in combination with conventional chemotherapy in patients with R/R AML and high‐risk MDS, and recommend starting doses for phase two studies of this combination. Secondary objectives were to define toxicities associated with this combination and assess therapeutic response.

Data are presented with percentage proportions for categorical variables and medians for continuous variables. The chi‐square test and Fischerʼs exact test were used to compare categorical variables. Survival estimates were calculated using the Kaplan‐Meier method and differences between curves were assessed using the log‐rank test. The overall survival (OS) time was calculated from the time point of study entry until death or last follow‐up. Relapse‐free survival (RFS) was calculated from the time point of CR/CRi until the time of relapse, death or the last follow‐up. Statistical analyses were performed using JMP software v.14.0.0 (SAS Inc, Cary, NC) and *P* ≤ .05 was considered statistically significant.

## 3 RESULTS

A total of 33 patients were enrolled in the study (30 AML, three MDS) and their clinical characteristics are summarized in Table 1. None of the patients had prior lenalidomide exposure.

**TABLE 1:** Clinical characteristics of patients

	Number of patients
Diagnosis	
MDS	3 (9%)
AML	30 (91%)
Secondary or therapy‐related	3 (9%)
Age, median, years (range)	57 (22‐64)
Gender	
Female	17 (52%)
Male	16 (48%)
ELN classification^a^	
Favorable	7 (22%)
Intermediate	9 (30%)
Adverse	15 (48%)
del(5q) present	2 (6%)
Molecular aberrations^b^	
*NPM1* mutation	7 (25%)
*FLT3‐*ITD mutation	7 (25%)
*TP53* mutation	5 (19%)
*RUNX1* mutation	3 (11%)
*ASXL1* mutation	2 (7%)
*CEBPA* mutation	1 (4%)
WBC at presentation, ×10^3^/μL, median (range)	3.7 (0.8‐27.3)
Peripheral blast %, median (range)	7 (0‐90)
Bone marrow cellularity, %, median (range)	60 (10‐95)
Bone marrow blast %, median (range)	38 (4‐92)
Number of prior therapies, median (range)	1 (1–4)
Previous hematopoietic cell transplantation	7 (21%)
MUD	4 (12%)
MSD	3 (9%)
Last CR duration	
Long (>1 y)	6 (18%)
Short (<1 y)	19 (58%)
Primary refractory	8 (24%)

Abbreviations: AML, acute myeloid leukemia; CR, complete remission; ELN, European Leukemia Net; MDS, myelodysplastic syndrome; MSD, matched sibling donor; MUD, matched unrelated donor; WBC, white blood cell count.

^a^ AML patients only (n = 30).

^b^ Sequencing was performed in 28 patients only.

### 3.1 Toxicity profile and MTD assessment

Toxicity data were collected for 33 patients and ≥grade three non‐hematologic adverse events are shown in Table 2. A total of eight DLTs (24%) were observed with four being hematologic DLTs. At dose level one, three patients were initially enrolled, of whom one of them experienced hematologic DLT (neutropenia). Three more patients were enrolled at this dose level, one had grade four confusion (requiring intensive‐care unit stay) which was likely multifactorial and not drug‐related. We subsequently enrolled three patients at dose level 2, one had prolonged grade four neutropenia and one patient died on day 5 of the study due to pulmonary hemorrhage after bronchoscopic procedure. Three more patients were enrolled at this dose level, one developed heart failure symptoms due to new onset reduced left ventricular ejection fraction, thought possibly due to anthracycline toxicity. Therefore, the dose was reduced back to dose level 1 with three more patients enrolled, two of whom had prolonged grade four neutropenia and one patient died from intracranial hemorrhage. This led to further de‐escalation to dose level −1, which delivered the same lenalidomide dose (25 mg daily) as the previous dose level, but both idarubicin and cytarabine were dose‐reduced (see Figure 1). The initial six patients enrolled at this level did not experience DLT, therefore this dose level was determined to be the MTD and recommended to pursue for the phase II dose level. Twelve subsequent patients were enrolled for pre‐planned dose expansion to further assess safety and tolerability. Only one patient experienced serious adverse event (SAE) with acute renal failure, septic shock and respiratory failure.

**TABLE 2:** Summary of grade 3 or higher non‐hematologic toxicities according to dose level

Toxicity description	Frequency				Any grade ≥3 toxicity by dose level		
	Grade 3 n (%)	Grade 4 n (%)	Grade 5 n (%)	Total n (%)	Level 2 (N = 6)	Level 1 (N = 9)	Level –1 (N = 18)
Febrile neutropenia	10 (30)	0	0	10 (30)	3	3	4
Rash	8 (24)	0	0	8 (24)	1	2	5
Sepsis	1 (3)	2 (6)	3 (9)	6 (18)	2	2	2
Elevated creatinine	3 (9)	1 (3)	0	4 (12)	2	1	1
Diarrhea	3 (9)	0	0	3 (9)	0	1	2
Confusion	1 (3)	1 (3)	0	2 (6)	0	2	0
Reduced LVEF	0	1 (3)	0	1 (3)	1	0	0
Intracranial hemorrhage	0	0	1 (3)	1 (3)	0	1	0
Pulmonary hemorrhage	0	0	1 (3)	1 (3)	1	0	0
Hypertension	1 (3)	0	0	1 (3)	0	1	0
Atrial fibrillation	0	1 (3)	0	1 (3)	0	0	1

Abbreviation: LVEF, left ventricular ejection fraction.

The most commonly observed non‐hematologic SAEs (ie, ≥grade three toxicities) were febrile neutropenia (30%), rash (24%), sepsis (15%) and acute kidney injury (12%). Death from any cause within 30 days of trial entry occurred in five (15%) of 33 patients. Death was a result of sepsis in the setting of active leukemia in three patients, pulmonary hemorrhage in one patient and progressive disease in one patient.

Overall, 15 out of 33 (45%) of patients were able to receive pre‐planned 21 days of lenalidomide therapy. In 18 patients, lenalidomide was held due to side effects with a median duration of therapy of 13 days (range, 5‐19 days of treatment). Among 18 patients treated at MTD level, eight (44%) were able to receive 21 days of treatment, while 10 patients had a median treatment duration of 14 days (range, 10‐19 days).

### 3.2 Clinical responses

Twenty‐nine out of 33 enrolled patients were evaluable for efficacy (Figure 1B). Response could not be assessed in four patients due to complications and mortality within the first 7 weeks of enrollment, with inability to perform a bone marrow disease assessment. Eighteen patients achieved CR (n = 14) or CRi (n = 4) with a total CR/CRi rate of 56% in the entire cohort. Among three MDS patients, one achieved CRi, one was refractory, and response could not be assessed in the other patient due to early death after bronchoscopic procedure. The CR/CRi rate was significantly lower for patients treated at dose level − 1 (MTD) when compared to patients treated at dose levels 1 and 2 combined (39% vs 73%, *P* = .04). In addition, all six patients who had a long duration of remission before trial enrollment (>1 year) achieved CR/CRi on this study, while CR/CRi rate was 47% for patients with shorter duration of last remission before study treatment (<1 year, n = 19) and 50% for patients with primary refractory disease (n = 6). There were two patients with del(5q), and both of them achieved CR. The median duration of CR/CRi was 5.1 months (range, 1‐93.5 months).

Seven patients enrolled in this study had AML relapse after allogeneic HCT. Among them, five (71%) achieved CR, with response durations of 1, 2.5, 4, 27 and 72 months. One patient experienced skin and liver graft‐vs‐host disease (GVHD) after lenalidomide therapy, requiring initiation of systemic steroids while another patient had mild skin GVHD. Only one patient received donor leukocyte infusion for relapsed disease after study treatment.

The median OS for the entire cohort was 6 months, and the median RFS for patients who achieved CR/CRi was 4.4 months (Figure 1C). The 1‐year and 2‐year OS were 24% and 10%, respectively. Patients who achieved CR/CRi after study treatment (ie, responders) had longer OS when compared to patients who were refractory to this regimen (ie, non‐responders) (median OS, 10 months vs 2 months, *P* = .001) (Figure 1C). Of the 18 patients who achieved CR/CRi, 10 underwent allogeneic HCT. The median OS and RFS of patients who had HCT were 11.4 and 7.3 months, respectively, and with a 1‐year OS of 40%.

### 3.3 Molecular profile and response

**FIGURE 2:**
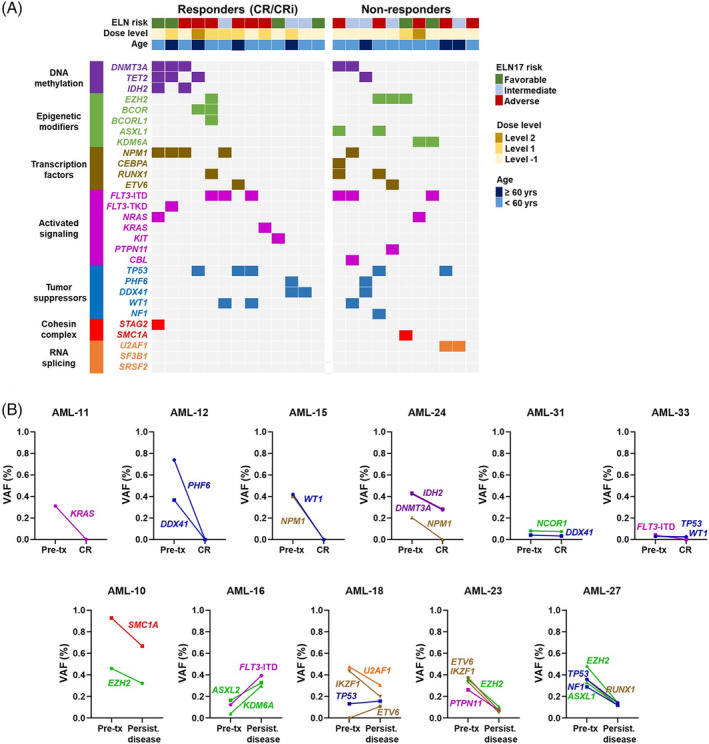
A, Oncoprint showing myeloid mutation profile of study patients stratified based on response to treatment. B, Serial pretreatment and posttreatment samples obtained from study patients were analyzed with targeted next‐generation sequencing panel. Variant allelic frequencies (VAF) of mutations detected before treatment and at the time of complete remission (CR) or persistent disease are illustrated [Color figure can be viewed at wileyonlinelibrary.com]

Next‐generation sequencing for recurrent somatic mutations was performed at study entry in 24 patients (Figure 2A). There was no clear association between mutation patterns and response to therapy. Twelve patients also had serial pre‐treatment and post‐treatment samples available for analysis (Figure 2B). Among seven patients with serial pre‐treatment and CR samples, three patients had no detectable mutations at remission suggesting eradication of mutated myeloid clones at the time of morphologic CR. Duration of response in these patients were 1, 28, and 31 months. An additional three patients had reduction in the size of their pre‐treatment clones and one patient did not have any detectable mutations at any time point. Among five patients who had serial pre‐treatment and post‐treatment refractory disease samples, though changes in the size of the clones was observed, all persisted after therapy.

## 4 DISCUSSION

Effective therapeutic options for patients with R/R AML are limited. Intensive chemotherapy is often less effective than in the front‐line setting and is associated with substantial toxicity. Several novel targeted therapies demonstrated significant clinical activity in R/R AML, but their use is restricted to specific biologic subtypes of AML such as patients with mutated *FLT3* or *IDH* genes. Therefore, the most common approach for fit patients with R/R disease is to achieve remission followed by allogeneic HCT. Historically, remission rates with salvage chemotherapy have been reported at 16%‐25%.^4^ In this phase one study, we found that lenalidomide given for 21 days in combination with 4‐day cytarabine and 3‐day idarubicin for patients with R/R AML had moderate toxicity with evidence of antileukemic activity in heavily pre‐treated patients as well as patients with short CR duration. The majority of patients enrolled in this study were refractory to the last treatment they received or had experienced relapse within 1 year of prior CR. This cohort was also enriched for patients with poor cytogenetic and molecular characteristics. The MTD of lenalidomide was 25 mg/d when combined with 4 days of 1 g/m^2^ cytarabine and 3 days of 8 mg/m^2^ idarubicin. However, it should be noted that majority of patients could not complete the full planned 21 days of lenalidomide therapy. The CR rate was 39% at this dose level and the most common toxicities were febrile neutropenia, rash and diarrhea. There was no apparent associated molecular pattern of response in this small cohort. Of the patients who achieved CR/CRi on this study, 56% (10 out of 18) were able to undergo HCT. Our observations are encouraging for further clinical development of lenalidomide in R/R AML with no targetable mutations.

We have previously reported that single‐agent lenalidomide had acceptable tolerability in patients with acute leukemia at doses up to 50 mg daily.^9^ This dose was used in single‐agent phase 2 studies of treatment‐naïve AML with CR rates reaching 30%.^13^, ^14^ However, studies investigating combinations of lenalidomide with other chemotherapy drugs reported marked hematologic toxicity at doses above 25 mg daily. One study initiated lenalidomide on days 6‐26 following 5‐day treatment with 1.5 mg/m^2^ cytarabine in R/R AML patients, and 10 mg daily dose was deemed to be the MTD.^11^ Similarly, studies combining lenalidomide with standard 7 + 3 induction either on days 1‐21 starting with induction,^18^ or on days 8‐21 following induction chemotherapy^27^ reported significant hematologic and other organ toxicity limiting dose escalation beyond 25 mg daily. Notably, another R/R AML study investigating lenalidomide combination with MEC salvage chemotherapy reported that high doses of lenalidomide (50 mg/d) can be safely administered if therapy was limited to days 1‐10.^12^ In this study, we could safely administer lenalidomide 25 mg/d with a dose‐adjusted chemotherapy regimen. However, administration of this dose beyond 10 days was associated with increased toxicity, which supports the 10‐day schedule of the previous study.

Preclinical data suggest that lenalidomide may sensitize leukemic blasts to cytotoxic chemotherapy.^8^ This was also observed in comparative analysis of paired marrow samples obtained before and after lenalidomide treatment of patients participated on phase one lenalidomide plus MEC study.^28^ Lenalidomide enhanced apoptotic priming in vivo and dynamic BH3 profiling assay was suggested as a biomarker to predict response to combination. Embarking on this encouraging mechanistic data, a recent phase two study of 222 newly diagnosed older AML patients compared standard 7 + 3 induction chemotherapy with or without lenalidomide at a dose of 20 mg daily on days 1‐21.^21^ Although the combination was well tolerated, addition of lenalidomide did not improve the CR/CRi rate, event‐free survival or OS. Given the importance of sequencing these drugs, future studies would be warranted to investigate the impact of pre‐treatment with higher doses of lenalidomide. It should also be noted that therapeutic activity of lenalidomide was more pronounced with prolonged treatment in other diseases (eg, myeloma). Therefore, a maintenance treatment strategy should also be considered in AML.

Lenalidomide has several immunomodulatory effects, including stimulation of innate, cellular and humoral responses.^6^ Initial reports of lenalidomide in AML relapsed after HCT showed promising antileukemic activity at a cost of severe GVHD.^9^ However, a recent phase one study in this setting demonstrated that a salvage regimen with 7 days of azacitidine followed by 25 mg daily lenalidomide on days 10‐30 (of a 30‐day cycle) was tolerable with acceptable rates of GVHD and 47% clinical response.^10^ It should be noted that patients in these series received either alemtuzumab or anti‐thymocyte globulin for GVHD prophylaxis as opposed to T‐replete allografts used in other lenalidomide maintenance studies. Additional studies are needed to optimize the lenalidomide containing salvage and maintenance regimens for post‐HCT AML patients.

In addition to its incompletely understood mechanism of action, the molecular subgroup of AML patients who may derive the most benefit from lenalidomide is not clearly defined. Given its efficacy in MDS with del(5q), prior studies focused on testing this drug in AML patients with del(5q) and showed only modest benefit.^13^ We reported single agent activity of lenalidomide in AML patients with isolated trisomy 13^9^, ^29^ and another brief report demonstrated clinical activity of lenalidomide in combination with hypomethylating agents in AML patients with inv(3).^30^ Due to the low frequency of these abnormalities in AML, lenalidomide clinical trials including the present study lack the statistical power to analyze individual karyotypic abnormalities. Similarly, our study and others could not identify a myeloid mutation signature predicting response to lenalidomide.^12^

This study has several limitations. Given the phase I nature of the study, data on efficacy of the combination regimen should be interpreted with caution. The trial population is heterogenous in regards to the spectrum of underlying mutational profiles and previous treatments. Analysis of serial samples demonstrated eradication of pre‐treatment mutations in patients who could attain CR. However, the NGS was performed in research laboratory and results may not have the sensitivity to document MRD negativity at remission.

Our data demonstrate that the combination of lenalidomide and cytarabine/idarubicin chemotherapy had clinical activity among patients with R/R AML. The combination could potentially be utilized as a bridge to HCT. The regimen was also effective for AML patients who experienced relapse after HCT, an area of major unmet need. It should be noted that the response rates were lower at MTD (dose level −1) and lenalidomide was discontinued in most patients beyond day 10. Therefore, a shorter course of lenalidomide therapy may increase the tolerability of combination. Further studies investigating combinations of lenalidomide with chemotherapy and targeted therapies, as well as maintenance therapy for patients in remission are warranted.

## CONFLICT OF INTERESTS

The authors declare no potential conflict of interest.

## AUTHOR CONTRIBUTIONS

Caner Saygin conceptualized the study, analyzed data, wrote the manuscript. Karilyn Larkin, James S. Blachly, Mitch A. Phelps, Shylaja Mani, Alison Walker, Ramiro Garzon, Sumithira Vasu, Katherine J. Walsh, Bhavana Bhatnagar, Rebecca B. Klisovic, Michael R. Grever, Guido Marcucci, John C. Byrd, William Blum, and Alice S. Mims conceptualized the study and collected data. Shelley Orwick, Apollinaire Ngankeu, and Charles T. Gregory performed sample sequencing and analysis. All authors edited and agreed on the final version of the manuscript.

## Data Availability

The data that support the findings of this study are available on request from the corresponding author. The data are not publicly available due to privacy or ethical restrictions.
